# Quality by Design of Pranoprofen Loaded Nanostructured Lipid Carriers and Their Ex Vivo Evaluation in Different Mucosae and Ocular Tissues

**DOI:** 10.3390/ph15101185

**Published:** 2022-09-24

**Authors:** María Rincón, Lupe Carolina Espinoza, Marcelle Silva-Abreu, Lilian Sosa, Jessica Pesantez-Narvaez, Guadalupe Abrego, Ana Cristina Calpena, Mireia Mallandrich

**Affiliations:** 1 Department of Materials Science and Physical Chemistry, Faculty of Chemistry, University of Barcelona, C. Martí i Franquès 1-11, 08028 Barcelona, Spain; 2 Departamento de Química, Universidad Técnica Particular de Loja, Loja 1101608, Ecuador; 3 Department of Pharmacy, Pharmaceutical Technology and Physical Chemistry, Faculty of Pharmacy and Food Sciences, University of Barcelona, Av. Joan XXIII 27-31, 08028 Barcelona, Spain; 4 Institut de Nanociència i Nanotecnologia IN2UB, University of Barcelona, 08028 Barcelona, Spain; 5 Pharmaceutical Technology Research Group, Faculty of Chemical Sciences and Pharmacy, National Autonomous University of Honduras (UNAH), Tegucigalpa 11101, Honduras; 6 Departamento de Estadística, Facultad de Ciencias Sociales, Universidad Carlos III, C. Madrid, 126, 28903 Madrid, Spain; 7 Department of Chemical and Instrumental Analysis, Faculty of Chemistry and Pharmacy, University of El Salvador, Ciudad Universitaria, 28040 Madrid, Spain

**Keywords:** design of experiment, porcine mucous membrane, ophthalmic tissues, permeation, nanostructured lipid carriers

## Abstract

Transmucosal delivery is commonly used to prevent or treat local diseases. Pranoprofen is an anti-inflammatory drug prescribed in postoperative cataract surgery, intraocular lens implantation, chorioretinopathy, uveitis, age-related macular degeneration or cystoid macular edema. Pranoprofen can also be used for acute and chronic management of osteoarthritis and rheumatoid arthritis. Quality by Design (QbD) provides a systematic approach to drug development and maps the influence of the formulation components. The aim of this work was to develop and optimize a nanostructured lipid carrier by means of the QbD and factorial design suitable for the topical management of inflammatory processes on mucosal tissues. To this end, the nanoparticles loading pranoprofen were prepared by a high-pressure homogenization technique with Tween 80 as stabilizer and Lanette^®^ 18 as the solid lipid. From, the factorial design results, the PF-NLCs-N6 formulation showed the most suitable characteristics, which was selected for further studies. The permeability capacity of pranoprofen loaded in the lipid-based nanoparticles was evaluated by ex vivo transmucosal permeation tests, including buccal, sublingual, nasal, vaginal, corneal and scleral mucosae. The results revealed high permeation and retention of pranoprofen in all the tissues tested. According to the predicted plasma concentration at the steady-state, no systemic effects would be expected, any neither were any signs of ocular irritancy observed from the optimized formulation when tested by the HET-CAM technique. Hence, the optimized formulation (PF-NLCs-N6) may offer a safe and attractive nanotechnological tool in topical treatment of local inflammation on mucosal diseases.

## 1. Introduction

Pranoprofen (PF) or 2-(5H-chromeno[2,3-b]pyridin-7-yl)propanoic acid is a non-steroidal anti-inflammatory drug (NSAID), which can be used as an effective and safe anti-inflammatory treatment option in ocular therapy [1,2,3]. It is usually indicated in chronic but non-bacterial inflammatory processes affecting the anterior segment of the eye and in the treatment of postoperative pain of cataract or strabismus surgery [4]. PF may also be used to control the inflammation and pain in the posterior segment, such as in the case of posterior chamber intraocular lens implantation, acute central serous chorioretinopathy, uveitis age-related macular degeneration or cystoid macular edema [5]. PF can also be used for acute and chronic management of osteoarthritis and rheumatoid arthritis [6,7]. PF inhibit COX-1 and COX-2 enzymes, and thus it blocks arachidonic acid being converted to eicosanoids and reduces prostaglandins synthesis. Although this drug has shown high anti-inflammatory and analgesic efficiency, and its side effects on the gastrointestinal tract are minimal, the pharmaceutical use of PF is limited due to its inadequate biopharmaceutical profile. PF has a short plasmatic half-life, low water solubility and is unstable in aqueous solution, particularly when exposed to light [6,8].

The application of NSAIDs through the mucosal tissues offers a set of advantages, e.g., it avoids the first pass metabolism, averts the risk of gastrointestinal disturbance, targeting only the areas of disease. The use of ophthalmic NSAIDs is the usual treatment to prevent and treat common ocular disorders inflammatory [9]. However, this conventional dosage form cannot be considered optimal in the treatment of ocular diseases due to the fact that most of the drugs is removed from the surface of the eye, following the instillation, by various mechanisms (tear dilution and tear turn over). Moreover, the relatively impermeable corneal barrier restricts the entry of foreign substances. As a result, less than 5% of the administered drug penetrates the cornea and reaches intraocular tissues [10].

Intranasal drug application is currently most used for the treatment of local inflammations such as common rhinitis, allergic rhinitis or for easing nasal congestion. The main advantage of nasal administration is the rapid drug absorption through this membrane due to the prevailing physical conditions of the nose, such as good blood circulation of the nasal mucosa, resulting in a quick local effect, and the rapid onset of action. Due to the fast local absorption, an unintentional systemic distribution of the active ingredient is prevented, eliminating the first pass metabolism and its side effects are avoided [11,12]. Other advantages are the high permeability of some drugs in the nasal epithelium and better compliance with the recommended treatment, improved comfort for the patient, and a sustained and prolonged effect compared to other delivery systems such as oral drug delivery systems [11].

The administration of drugs through the oral mucosa, particularly the buccal and sublingual mucosa, has been attracting great interest. The main advantage of using the buccal route is the direct access of drugs to the systemic circulation by the internal jugular vein, eliminating the hepatic first-pass metabolism and mitigating possible side effects [13,14]. Nevertheless, buccal drug delivery suffers from some disadvantages such as low permeability and a smaller absorptive surface area, in contrast to the high absorptive surface area of the small intestine [15,16].

Vaginal drug delivery treats or prevents diseases and allows a controlled or enhanced drug absorption with the advantage of a systemic circulation delivery of drugs avoiding the hepatic first-pass effect and gastrointestinal interferences while assuring, in accordance with the selected formulation, stable and/or high local concentrations and reduced, enhanced and/or controlled systemic absorption [17].

In an attempt to overcome, the biopharmaceutical profile and improve the permeability of drugs through the tissues, nanostructured Lipid Carriers (NLCs) are one of the colloidal systems that have been most widely studied due to their characteristics such as small particle size, biocompatibility, prolonged release, drug protection, and incorporation of both hydrophilic and lipophilic drugs [18]. In ocular delivery, conventional ophthalmic formulations are effortlessly drained through the nasolacrimal pathway, while nanoparticles are cleared more slowly and therefore can release the drug upon interaction with the cornea over a longer time period [10].

Taking into account all these considerations, the main purpose of the work in question here was to target PF-loaded NLCs (PF-NLCs) in mucosal tissues (buccal, sublingual nasal, vaginal, cornea and sclera) with controlled release effect, enhancing the contact of the PF and improving its mucosal tissues retention, thus to increase the anti-inflammatory and analgesic. For this purpose, a factorial design and detailed statistical studies shed light on the most adequate lipid and led to the optimized formulation with physicochemical properties to permeate the mucosae in a competent way. Taking into consideration that factorial designs provide the maximum information from the least number of experiments [19], a 2^3^ +star central composite factorial design was applied to study the main effects and interactions of three factors: PF concentration (cPF), the concentration of the solid lipid in relation to the liquid lipid (cSL/cLL) and the concentration of Tween^®^ 80 (cTW), on dependent variables: average particle size (Z-Ave), zeta potential (ZP) and polydispersity index (PI), and encapsulation efficacy (EE). From a total of 16 formulations obtained by factorial design, an optimized formulation was selected to carry out additional studies. These formulations were characterized for their morphology rheological and extensibility. The physical stability of the optimized formulation was also evaluated, as well as the ex vivo permeation profile through porcine mucosal tissues (buccal, sublingual nasal, vaginal, cornea and sclera) and the in vitro tolerance study were assessed.

Design of Experiments is extensively used for the implementation of Quality by Design (QbD) in research and industrial settings. In QbD, formula (product) and process understanding are the key enablers of assuring quality in the final product [20]. QbD is not only a guide, but it also provides a systematic approach to drug development, focusing on quality through the application of analytical and risk management methodologies for the design, development and manufacture of new drugs. Likewise, it maps the influence and interaction of various formulations and operating parameters on process performance [21].

## 2. Results

### 2.1. Lipid Screening

The PF was dissolved in different solid lipids (SL) and liquid lipids (LL) in order to determine the components of the lipid phase before producing the NLCs containing PF. Therefore, components of the lipid phase were determined by evaluating the solubility of PF in 14 different lipids as shown in Table 1. Selected liquid lipids included LAS (PEG-8 Caprylic/Capric Glycerides), Castor oil, rose mosqueta oil, plantacare oil, Jojoba oil and Miglyol^®^ 812, and isopropyl myristate. The solubility of PF was also evaluated in solid lipids such as stearic acid, Precifac^®^ ATO, Compritol^®^ ATO 888, Precirol^®^ ATO 5, Lanette^®^ 18, Geleol^®^ and Gelucire^®^. Following on from this, PF (0.1 to 1% of PF was dissolved in different SL and LL or physical mixtures of them (PF, 1.5 to 3%, with regard to total lipid). PF in either physical mixtures or lipids was heated at about 10 °C above the melting point of the SL. The samples obtained were then observed to verify the presence/absence of insoluble drug crystals. The next step was that the mixtures were cooled down to room temperature (RT) for solidification, and this was followed by the analysis of the lipids and PF by Differential Scanning Calorimetry (DSC) to evaluate non-solubilized PF. The analysis was carried out under the conditions described below in the assessment of the lipid matrix crystallinity. Another condition that we imposed is that any resulting solid lipids that were “soft” or “semi-solid” at room temperature were not considered for the design of the NPs (Table 1 and Table 2).

Based on the results from the lipid screening, the selected lipids for the preparation of the PF-NLC formulations were Castor oil, LAS and Lanette^®^ 18. In previous studies, we formulated the NLCs with Castor oil, LAS and Precirol^®^ ATO 5, which were chosen and studied [8,18].

### 2.2. Design of Experiments

The PF-NLC formulations were optimized by means of a 2^3^ + star central composite rotatable design (Table 3 and Table 4). The main effects and interactions of the independent variables, such as concentration of solid lipid with regard to liquid lipid (cSL/LL), concentration of PF, and concentration of Tween 80^®^ (cTW) were investigated on the mean particle size (Z-Ave), polydispersity index (PI), zeta potential (ZP) and encapsulation efficiency (%EE). A total of 16 experiments are summarized in Table 4.

Four statistical techniques are presented to reveal and visualize the relationship between the aforementioned trials, the independent variables (cLS/L), (cPF), (cTW) and the dependent variables (ZP (mv), Z-Ave (nm), Pl and EE (%)).

#### 2.2.1. Principal Component Analysis (PCA)

PCA is a classic multivariate analysis technique used to represent n trials in a synthetic variables space known as *principal components*. This method permits the better visualization of the most representative variables and trials’ behavioural patterns of each component. For further details, see the work of Peña [22].

The stages of PCA consist of: (i) plotting and measuring the percentage of explained variability of each principal component, (ii) deciding the number of components, (iii) representing and interpreting results through biplots.

**Figure 1:**
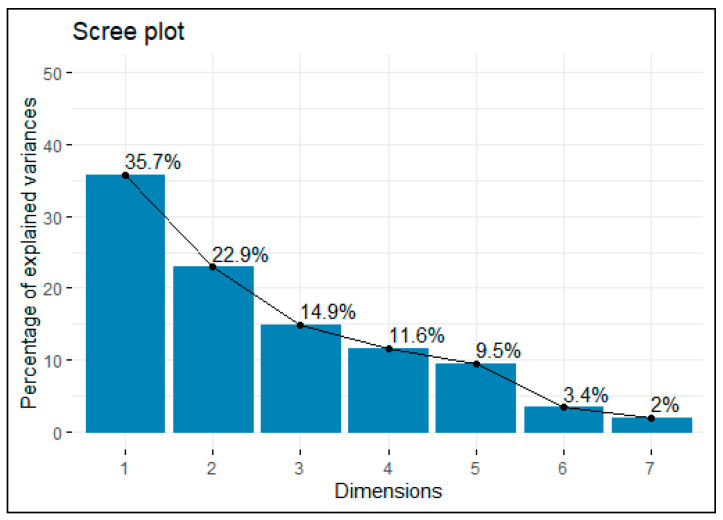
Scree plot of the variance of the components.

Figure 1 shows a scree plot of the variance of each principal component, and Table 5 shows a descriptive summary of the importance of all the components. The decomposition into 3 first principal components explain 74% of the information. Under the elbow criterion, this threshold might be considered high enough to draw conclusions from it.

Table 6 presents a correlation matrix between variables and principal components. We consider a high representation of variables whose correlation with the component is higher than 70%.

**Figure 2:**
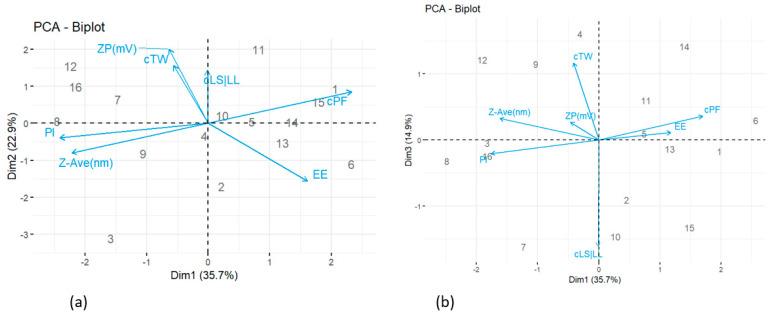
Biplots of (**a**) the First and Second Principal Component; (**b**) the First and Third Principal Component.

Figure 2a is a biplot for the first and second principal components (PC1–PC2). The *x*-axis (PC1) is represented mainly by cPF, Z-Ave (nm) and Pl. The latter has a negative strong correlation with variables cPF and Z-Ave (nm), specifically the greater the values of cPF, the lower the values cPF and Z-Ave (nm) and vice versa. In particular, trial 8 has the highest levels of Pl and Z-Ave (nm), the very opposite of trials 1, 14 and 15.

The most represented variables on the y-axis (PC2) are ZP (mv) and EE, both are inversely related. Trials 12, 16, 7 and 11 have the largest concentrations of ZP (mv); and thus, the lowest EE, in contrast to trial 2 which has the maximum EE (%).

Trials 4, 5 and 10 showed mean concentrations of both types of variables, dependent and independent.

Figure 2b is a biplot for the first and third principal components (PC1-PC3). The *x*-axis (PC1) is weakly represented by cLS/LL and cTW, both variables are inversely correlated. Trials 2 and 10 have the largest levels of cLS/LL and the lowest of TW. In contrast, trials 4 and 9 have the highest TW and cLS/LL. In addition, the *y*-axis (PC3) has no correlations larger than 70%.

#### 2.2.2. Boxplots

**Figure 3:**
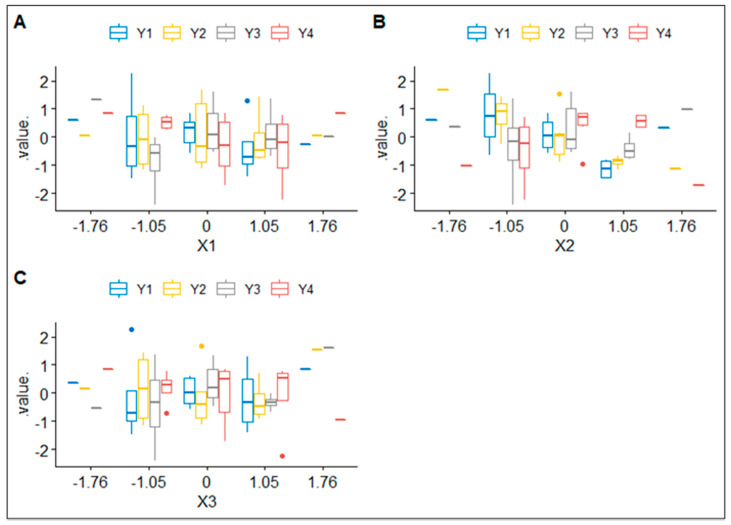
Boxplots of independent variables (cLS/LL, cPF and cTW) according to their corresponding dependent variables (Z-Ave (nm), Pl, ZP (mV) and EE (%)), represented in subfigures (**A**–**C**) respectively. X1, X2 and X3 denote cLS/LL, cPF and cTW, respectively, and Y1, Y2, Y3 and Y4 denote Z − Ave (nm), Pl, ZP (mV) and EE (%), respectively.

Figure 3 shows three boxplots with cLS/LL, cPF and cTW represented on each *x*-axis and compared to their corresponding Z-Ave (nm), Pl, ZP (mV) and EE (%).

When independent variables take extreme values (−1.76 or 1.76), the interquartile range is extremely small, in fact, dependent variables are barely influenced. However, when independent variables take more intermediate values, the dependent ones present a much higher dispersion; thus, they become more sensitive to changes in cLS/LL, cPF and cTW.

#### 2.2.3. Multivariate Analysis of Variance

The multivariate analysis of variance (MANOVA) is an extension of the ANOVA which includes two or more dependent variables instead of only one. It is used to examine the effects of factor independent variables cLS/LL, cPF, cTW on continuous dependent variables Z-Ave (nm), ZP (mV), Pl and EE (%).

Table 7 shows the results of Manova. cPF is the only statistically significant variable at the 5% level. Therefore, the means of the levels of the factor cPF are significantly different from each other in all the responses (dependent variables).

#### 2.2.4. Four-Dimensional Graphics

**Figure 4:**
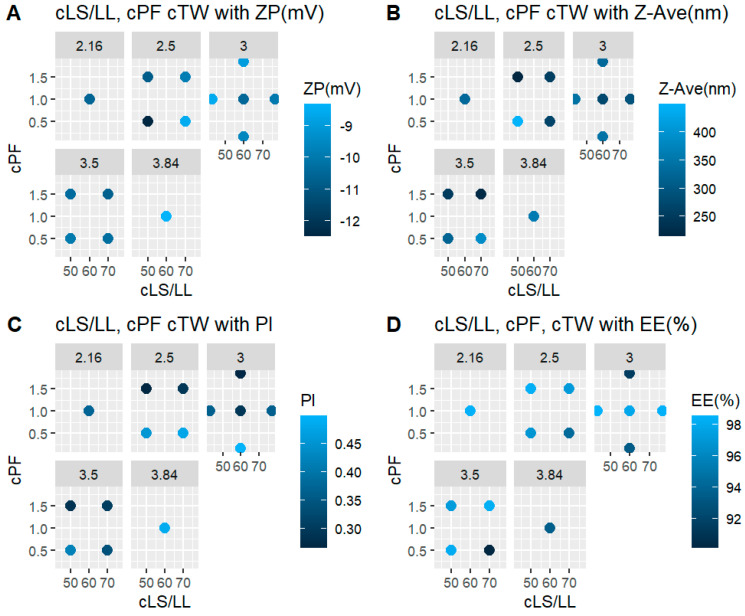
Four-dimensional plots of dependent and independent variables: (**A**) relationship between the independent variables with the zeta potential; (**B**) relationship between the independent variables with the mean particle size; (**C**) relationship between the independent variables with the polydisopersity index; and (**D**) relationship between the independent variables with the encapsulation efficiency.

Figure 4 shows four (A, B, C and D) four-dimensional (4D) plots that reveal the relationship of the independent variables (cLS/LL, cPF and cTW) with the dependent variable (ZP (mV). In this case, cLS/LL is represented on the *x*-axis and cPF on the *y*-axis, both variables plotted by each one of the 5 levels of cTW, and coloured by the intensity of ZP (mV), Z-Ave (nm), Pl and EE (%), respectively.

Case A of Figure 4 shows that intermediate values of cLS/LL, cPF and cTW stimulate a medium concentration of ZP (mV), but low values of cLS/LL and cPF with intermediate values of cTW decrease substantially the concentration of ZP (mV).

Case B of Figure 4 shows that intermediate values of cLS/LL, cPF and cTW generate a medium concentration of Z-Ave (nm), in contrast to intermediate levels of cLS/LL and cPF with high levels of cTW that influence Z-Ave (nm) to range from 300 to 400 approximately. Low levels of cLS/LL and cPF with intermediate levels of cTW substantially increase the concentration of Z-Ave (nm). Moreover, high levels of cPF with intermediate levels of cTW tend to decrease the concentration of Z-Ave (nm).

Case C of Figure 4 shows that intermediate levels of cLS/LL, cPF and cTW cause a low concentration of Pl. However, intermediate values of cLL/LL and cPF with high levels of cTW decrease the concentration of Pl. Low levels of cLS/LL and cPF with intermediate levels of cTW cause a medium concentration (0.36–0.45 approximately) of Pl. Moreover, high levels of cPF with intermediate values of cTW decrease the concentration of Pl, while low values of cPF with intermediate values of cLS/LL and TW increase the concentration of Pl.

Case D of Figure 4 shows that intermediate values of cLS/LL and TW generate a medium-high concentration (69–98) of EE (%). However, intermediate values of cLS/LL and cPF with high values of cTW decrease the concentration of EE (%). Low levels of cLS/LL and cPF with intermediate values of cTW cause a medium concentration of EE (%). Intermediate values of cLS/LL and cPF with low values of cTW increase the concentration of EE (%) whilst with high values of cTW, the concentration of EE (%) decreases.

### 2.3. Physicochemical Characterization

#### 2.3.1. Particle Size, Zeta Potential and Encapsulation Efficiency

The developed PF-NLCs formulation selected was the number 6 (see Table 4), which exhibited a Z-Ave around 214.20 nm, a negative surface charge with ZP values around −10.81 mV, and PI values of 0.266 that indicate a monomodal distribution (lower than 0.3). The percentage of EE shows values around 98% indicating that PF is inside of the NLCs. To evaluate the presence of large particles, the Z-Ave for PF-NLCs was measured by Laser Diffraction (LD) using a Mastersizer Hydro 2000 (Malvern Instrument Ltd., Malvern UK). After one day of production, PF-NLCs-N6 showed values of 0.087 d (0.1), 0592 d (0.9) and 0.199 d (0.5), which indicated that more than 50% (V) of the particles were smaller than 199 nm in all cases.

#### 2.3.2. Morphological Characterization

**Figure 5:**
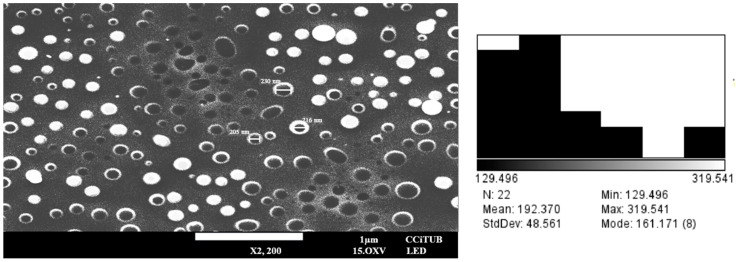
Scanning Electron Microscopy image of PF-NLCs-N6 and the related histogram.

The morphology of the optimized PF-NLCs was determined by SEM. The results showed spherical and no aggregated particles (Figure 5); the mean particle size of the sample was 192.37 nm ± 48.56 nm, confirming the results obtained by Photon correlation spectroscopy.

### 2.4. Rheological Studies

**Figure 6:**
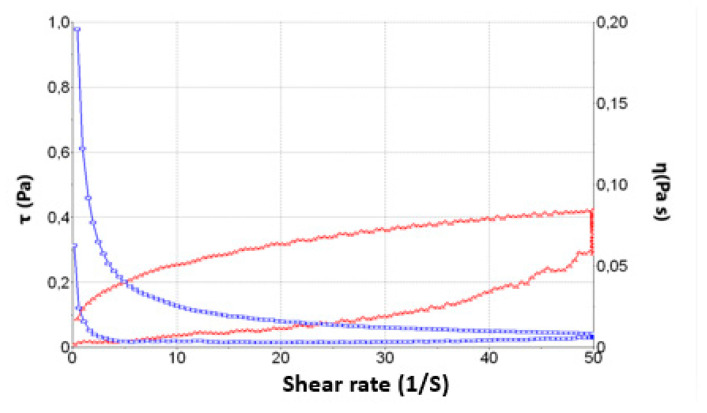
Rheological behaviour of PF-NLCs-N6.

The mathematical Cross best fitted the data. Figure 6 shows the rheological behaviour of PF-NLCs-N6. In addition, the viscosity at 25 °C from the constant velocity period of 50 s^–1^ was 0.77 × 10^2^ ± 0.76 mPa·s, demonstrating repeatability between samples (*n* = 3) in the rheological results.

### 2.5. Extensibility (Spreadability)

**Figure 7:**
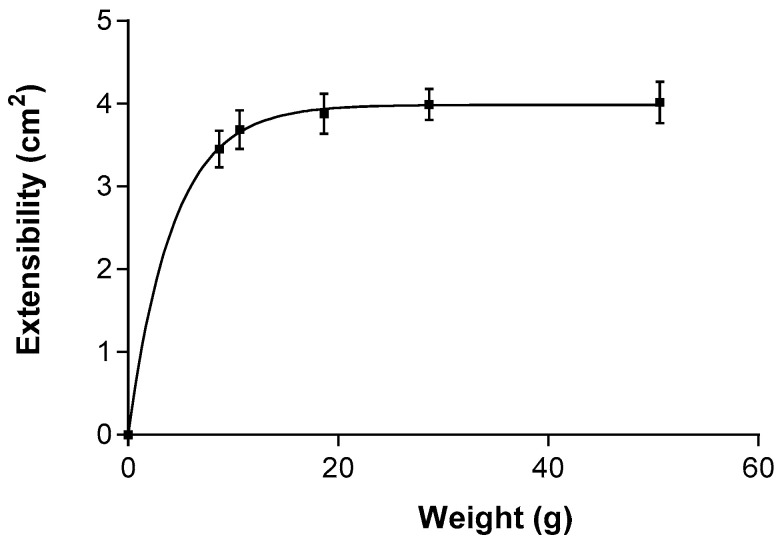
Fitting of PF-NLCs-N6 for the extensibility at 25 ± 2 °C; 60% ± 5% RH (*n* = 3). The extensibility followed a first-order model.

PF-NLCs showed a first-order model (Figure 7). The maximum extensibility (Ymax) obtained by the mathematical modelling was 2.023 ± 0.088 cm^2^ and the constant (K) was 0.138 ± 0.022 g^−1^. The data are expressed as mean ± standard deviation of three replicates (*n* = 3).

### 2.6. Stability Studies

**Figure 8:**
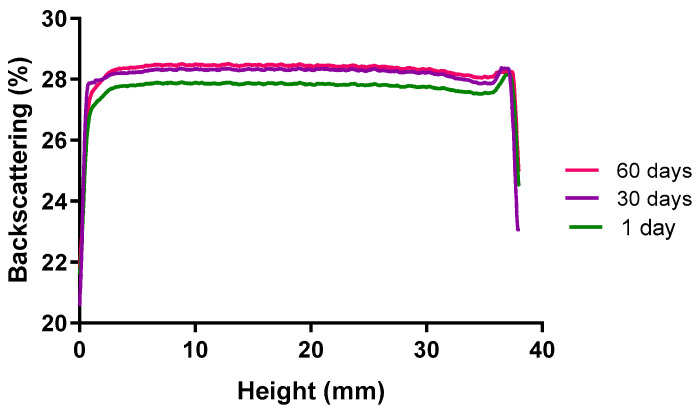
Backscattering of the PF-NLCs-N6 at different stability time points stored at 25 °C (*n* = 3). The left side of the curve represents the bottom of the vial, and the right side is the behaviour in the upper part of the vial.

The prediction of the accelerated stability of the PF-NLCs-N6 was evaluated for 60 days and measured to assess their short-term stability. It was studied after 1, 30 and 60 days of storage at 25 °C. Turbiscan^®^ Lab was used to observe the destabilization processes such as the alteration in the migration speed of the particles (vertical sections of the graph) and the variation in size (horizontal section of the graph) (Figure 8).

The migration of the particles to the top part of the cell leads to a drop in the concentration in the bottom part. This is shown as a drop in the backscatter signal (negative peak) and in reverse for the phenomena that happen in the top part of the vial. It is considered that the backscatter profile with a deviation of ± 5% does not present significant variations in particle size. Variations of ± 10% indicate that the formulation is unstable. It was observed that the back-scattered light profile did not show fluctuations greater than 5%, which indicates that our formula remained stable stored at 25 °C.

In addition, physical-chemical properties were measured over 90 days of storage at 25 °C and 4 °C, and the results are shown in Table 8 and Table 9.

**Figure 9:**
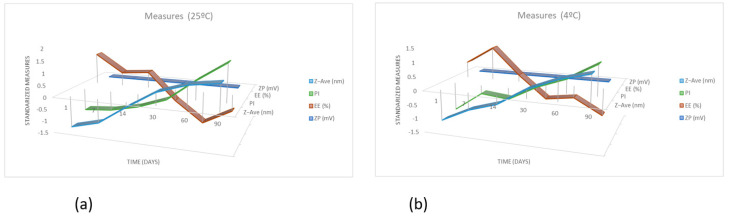
Graphics of the evolution of Z − Ave (nm), ZP (mV), EE (%) and PI over 90 days of PF-NLCs-N6 at 25 °C (**a**) ± and 4 °C (**b**) (*n* = 3).

The graphics of Figure 9a,b show the evolution of Z-Ave (nm), ZP (mV), EE (%) and PI over 90 days when the temperature is 25 °C and 4 °C, respectively. Z-Ave (nm) and ZP (mV) do not show relevant changes across time. However, the behaviour of EE (%) in the first and last days of the trial at 25 °C is in contrast to the trial of 4 °C. PI has a sudden rise, in comparison to the other trial, on the fourteenth day, when the temperature is 4 °C.

### 2.7. Ex Vivo Permeation Studies in Porcine Mucosal Tissues

We estimated the permeation parameters starting from the ex vivo permeation studies on different mucosal tissues, including six replicates per tissue. Table 10 reports the results obtained for flux, lag-time, partition and diffusion coefficients as well as the permeability coefficient. Cornea showed the greater flux, whereas similar values were observed for the rest of the mucosae (buccal, sublingual, nasal, vaginal and scleral tissue).

Regarding the lag time Tl (h), which is the time for the drug to reach the steady state, very quick absorption took place in the cornea. Nasal and vaginal mucosae showed intermediate lag-times, and buccal, sublingual and scleral tissue took a long time to reach the steady-state. This is probably correlated to the diffusion coefficient, which apparently is the major mechanism involved in the permeation (versus the partition coefficient), which in turn, may be due to the structural differences of the tissues tested [23,24]. The permeability coefficients were also similar for the four mucous membranes and about twice for the cornea.

Table 11 shows the recovery of PF from each tissue studied after the application of PF-NLCs-N6. In addition, Table 12 reports the amount of PF remaining in the tissues, expressed per surface area of mucosa exposed and per gram of tissue.

Table 13 shows the plasma concentration that would be achieved in the steady-state after the application of PF-NLCs-N6 on 1 cm^2^ of porcine mucous membrane (buccal, sublingual, nasal and vaginal) and ophthalmic tissues (sclera and cornea). The predicted Css values were below the therapeutic concentrations in plasma 4.89 ± 1.29 µg/mL for young subjects and 10.19 ± 2.43 µg/mL for elderly subjects [25].

### 2.8. Hen’s Egg Test on the Chorioallantoic Membrane (HET-CAM)

**Figure 10:**
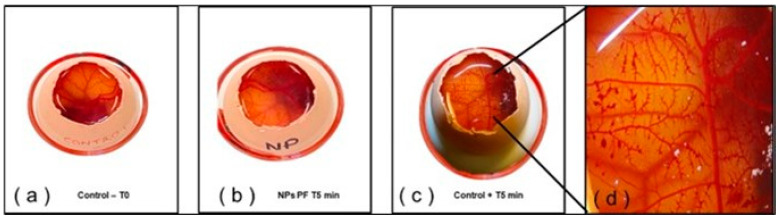
HET-CAM test: (**a**) Saline solution (Negative control-free PF-NPLCs); (**b**) PF-NPLCs-N6; (**c**) 0.1 N sodium hydroxide (Positive control); and (**d**) lesions caused by the positive control.

To establish ocular tolerance, an HET-CAM in vitro test was applied. PF-NLCs-N6 and free PF-NLCs were tested in the CAM of 3 eggs per formulation, to determine the possible rapid irritation reaction. The addition of 0.1 N NaOH (positive control) produced intense vasoconstriction and haemorrhage. In contrast, 0.9% NaCl (negative control) produced no reaction over the time tested. Similarly, the application of PF-NLCs-N6 onto the CAM did not expose any sign of intolerance or vascular alteration. Considering Figure 10, it is possible to confirm the suitability for ocular administration.

## 3. Discussion

AINEs in pig mucous and ocular tissues can be studied to learn a lot about veterinary and human medicine. Pig eyes are useful in comparative studies due to their many parallels to human eyes, including possessing a holangiotic retinal vasculature, cone photoreceptors in the outer retina, no tapetum, and they have a similar scleral thickness, making them very useful in comparative research [26]. When compared to other animal models, porcine buccal mucosa has been employed most frequently as a representative model for human buccal mucosa historically by multiple prestigious research groups worldwide [27,28].

The mouth can be affected by significant inflammatory processes because of localized or systemic diseases manifest in various types of buccal sores, such as lichen planus or canker sores, conditions that commonly present with inflammation and pain. In addition, although the oral cavity has its own bacterial flora, a qualitative and quantitative imbalance of this ecosystem leads to infections that produce inflammatory processes [29]. Regarding porcine vaginal mucosa, comparative studies with human vaginal mucosa reveal similarities in the morphology between species, both possess a nonkeratinized, stratified, squamous surface epithelium. The lipid composition of vaginal epithelium from pigs and humans shows similar concentrations of lipids, including ceramides, glucosyl ceramides, and cholesterol, which are the key permeability barrier components. This similarity in barrier lipids is reflected functionally in the data from permeability studies [30]. Concerning the nasal mucosa, morphological similarities between porcine and human mucosa, combined with ethical considerations when using pig model for studies, are reasons to continue with in vivo nasal absorption studies in various animal models that are correlated with human data and that have recently been presented [31].

A drug’s capacity to pass through mucous membrane and ocular tissues, such as PF, depends on both its physicochemical characteristics and the pharmaceutical formulation [32,33]. To reduce inflammation, it is essential to make sure the medicine is delivered to the site of action at therapeutic concentrations, and that these concentrations remain there for a long time. In this regard, using NLCs is a judicious substitute for using traditional medications. PF-NLCs-N6 was selected as the optimized formulation based on the qualities observed in the factorial design (Table 4). It is common knowledge that smaller particles adhere more strongly to surfaces such as tissues. SEM pictures attest to the effectiveness of the preparation strategy. The ZP is a measure of the particle charge which can influence both the stability of the particle and its mucoadhesion. Aggregation is prevented by electrostatic repulsion between particles with the same polarity of electrical charge. There was a net negative charge in every formulation. Although values of about ± 10 mV in zeta potential value may be considered too low to ensure stability, the results of the stability study demonstrated the PF-NLCs-N6 was a stable system. The stability is probably maintained by the addition of Tween^®^ 80 in the formulation, since non-ionic surfactants stabilize the colloidal systems by the steric effect instead of electrical repulsion between particles [34]. Concerning the relationship between mucoadhesion and the surface particle charge, it is accepted that the presence of a cationic surface charge in the lipidic nanocarrier may increase the residence time compared to negatively charged nanocarriers, because the positively charged systems interact with the corneal epithelium and the mucins from tears fluid and the conjunctiva, which they are all negatively charged [35,36].

The rheological and viscosity results suggest that the formulation can be easily and gently applied, resulting in low values of viscosity. The formulation was evaluated for extensibility or spreadability, which fitted first-order kinetics. The results suggest that the formulation is suitable for use as eyedrops (Figure 7).

The PF-NLCs-N6 showed no signs of destabilisation after 60 days at 25 °C as was shown by the backscattering profile having variations of less than 10%, indicating that the formulation was stable; contrarily variations greater than 10% would have signified an unstable formulation. Additionally, no signals of creaming, sedimentation, flocculation or coalescence were found. This system allows the predicting of the instability processes of NPs sooner than with other techniques [37]. These results are in line with previous studies with PF-NLCs with Precirol^®^ ATO 5 as solid lipid [8,18]. The high physicochemical stability of the formulation was also confirmed by no changes in the physical and chemical parameters Z-ave PI, ZP and EE, and the stability is also supported by negligible changes in the drug content (EE). Based on these results, we concluded that the nanoparticles are stable for at least 90 days at the different storage conditions studied since no apparent agglomeration occurred. In addition to this, the drug seems to have high compatibility with the formulation’s components.

An ex vivo permeation assay was carried out using the selected formulation, ex vivo permeation studies provide useful information to predict in vivo behaviour of the formulation [38,39]. It is known that the permeation of NSAIDs through the cornea is higher than through the scleral tissue and other ocular structures [23,40]. We observed the same trend for the flux and permeation coefficient in the permeation tests. The flux is the diffusion rate of pranoprofen into the eye and as, already mentioned, these differences may have their origin in different anatomical structures. This is the opposite of other studies that suggest that despite the fact that both tissues have a similar thickness (900µm), the sclera is ten times more permeable than the cornea [41]. The same pattern was observed for the permeability coefficient, the greatest value of Kp was found in the cornea. In addition, the Tl in corneal tissue is lower compared to the buccal mucous (minutes versus several hours). Taking into account that the Tl is representative of the time required for the drug to reach a steady state, the results suggest that PF-NLCs-N6 is rapidly absorbed in the cornea with a high diffusion. This is a desirable situation in anti-inflammatory drugs, which are aimed at achieving rapid action. Moreover, Tween^®^ 80 as a surfactant could expand the cornea membrane by potentially increasing its permeability, which could be key in the drug delivery of PF into the eye and even to the posterior segment of the eye [39]. The higher amount of PF retained in the cornea than the sclera (about 2.5-fold) favours a deposit of PF that could act over a longer time. Other researchers also investigated the amount of PF retained in ophthalmic tissues from nanostructured formulations and observed a lower amount of drug retained in the membranes. A similar study with human skin and PF-NLC with Precirol^®^ ATO 5 as solid lipid showed a Qr of about 24.29 µg/cm^2^/g [18]. Other studies carried out with nanoparticles loading an NSAID drug with similar results in the physicochemical characterization also resulted in lower drug retention [2,23]. However, in both cases, the nanoparticles consisted of poly D, L-lactic-co-glycolic acid (PLGA), which suggests that the formulation strongly impacts the permeation capacity of PF through the ophthalmic tissues.

Among the studied tissues, sublingual mucosa exhibited the highest amount of PF retained in the membrane, and the vaginal mucosa the lowest. Finally, the nasal and buccal mucosae showed intermediate values. The high amount of PF deposited in the sublingual mucosa suggests the PF-NLCs-N6 as a promising vehicle for the local delivery of PF in the sublingual mucosa.

From all the mucosae studied, the predicted plasma levels that were obtained at the steady state would be below the therapeutic concentration in plasma, considering when PF-NLCs-N6 is applied on 1 cm^2^ of tissue, meaning that no systemic effect would be observed and hence confirming the safety of formulation topically applied, while having a local analgesic and anti-inflammatory effect.

By observing negative changes that take place in the chorioallantoic membrane of the egg after being exposed to test substances, it is possible to identify compounds that may cause irritation (Figure 10). PF-NLCs-N6 is classified as a non-irritating drug at the ocular level, as shown in investigation. These findings are consistent with information obtained by other authors who also developed nanoparticles for ocular administration [42].

These results, which are in accordance with our earlier research, offer enormous opportunities for the local treatment of numerous inflammatory illnesses in humans or pigs, while pranoprofen side effects will be reduced. Despite this, additional research is needed to develop a pharmaceutical dosage form that makes its administration more convenient and effective.

## 4. Materials and Methods

### 4.1. Materials

Pranoprofen (2-(5H-chromeno[2,3-b]pyridin-7-yl)propanoic acid) (CAS 52549-17-4) was gratefully provided by Alcon Cusi (Barcelona, Spain). Tween^®^ 80 (Polyethylene glycol sorbitan monooleate) and Castor oil (Ricinus communis L.) (CAS 8001-79-4) were acquired from Sigma-Aldrich Química (Barcelona, Spain). Lanette^®^ 18 (stearyl alcohol) was acquired from Cognis (Dusseldorf, Germany). LAS (PEG-8 Caprylic/Capric Glycerides), Precifac^®^ ATO (cetyl palmitate), Compritol^®^ ATO 888 (glyceryl behenate), Precirol^®^ ATO 5 (glycerol mono, di and tripalmitostearate), Geleol^®^ (Glyceryl Monostearate) and Gelucire^®^ 44/14 (Polyoxylglycerides) were supplied by Gattefosse (Saint-Priest, France). Rose mosqueta oil, Plantacare oil, Jojoba oil and Miglyol^®^ 812 were provided by Roig Farma-Fagron (Tarrasa, Spain), isopropyl myristate was supplied by Merck (Darmstadt, Germany), and stearic acid (a saturated fatty acid (of C18) was provided by Croda Industrial Specialities (Nettetal, Germany). MilliQ water (resistivity > 18 MOhm.cm) was obtained by a MilliQ^®^ Plus System lab supplied. Phosphate buffer saline (PBS) pH 7.4 (tablets) was purchased from Sigma (Germany) and prepared as indicated by the manufacturer. All the other reagents and chemicals used in this research were of analytical grade.

### 4.2. Lipid Screening

Based on a list of suitable lipids (solid and liquid), the solubility of PF in different solid lipids (SL) and liquid lipids (LL) was performed to determine the components of the lipid phase before producing the NLCs containing PF.

### 4.3. Development of NLCs

A high-pressure homogenization technique was used to produce the NLCs, as described beforehand [18]. The lipid phase, which was 5% *w*/*w* of the total amount of formulation, consisted of Castor oil, the liquid lipid (LL) and Lanette^®^ 18 (75:25), the solid lipid (SL). The lipid phase in conjunction with PF was melted at 85 °C in a water bath resulting in a homogeneous lipid solution. An aqueous solution of Tween^®^ 80 as surfactant was heated in parallel at the same temperature and then added to the lipid phase, obtaining a primary emulsion by an Ultra-Turrax T25 (IKA, Staufen, Germany) at 8000 rpm for 45 s. Next, the emulsion was homogenized by a high-pressure homogenizer (Homogeniser FPG 12800, Stansted, UK) at 800 bar and 85 °C in three homogenization cycles. The NLCs were formed once the lipid recrystallized during the cool-down to room temperature. The NLCs were characterized for Z-Ave, PI, ZP and %EE, as described before [8].

### 4.4. Design of Experiments

A design of experiments (DoE) was performed to optimize formulation parameters. A central composite factorial design 2^3^ (containing 2 replicated centre points, 8 factorial points and 6 axial points) was developed using the statistical program Statgraphics Centurion XVI.II^®^ v. 16.2.04 (Warrenton, VA, USA).

In this section, we present some statistical techniques to reveal and visualize the relationship between the aforementioned trials with the independent variables (cLS/L), (cPF), (cTW) and the dependent variables (ZP (mv), Z-Ave (nm), Pl and EE (%)). The selected techniques for this analysis are principal component analysis (PCA), MANOVA, boxplots and four-dimensional plots performed with R, statistical software, version (4.0.0).

Considering the fact that trials are measured by different experimental units, the first two techniques are applied based on standardized input data (by subtracting the mean and dividing by the standard deviation) so that variables are treated equally, and the outcomes are not influenced by the units of measurement.

### 4.5. Physicochemical Characterization

#### 4.5.1. Particle Size and Zeta Potential

Photon correlation spectroscopy (PCS) technique by a Zetasizer Nano ZS (Malvern Instruments, Malvern, UK) [8,18], was used to determine the Z-Ave and PI of PF-NLCs. Samples were diluted (1:20 *v*/*v*) with Milli-Q water and measurements were carried out in triplicate at 25 °C in disposable quartz cells. In addition, the ZP was determined by electrophoresis laser-Doppler using the same instrument, with prior dilution in Milli-Q water (1:10 *v*/*v*) [19].

Furthermore, the particle size of PF-NLCs-N6 was measured by Laser Diffraction (LD) using a Mastersizer Hydro 2000 (Malvern Instrument Ltd., Malvern, UK) to evaluate the presence of large particles [18,34]. The volume distribution method served to determine the diameter values by Mie analysis including d (0.1), d (0.5), and d (0.9). The diameter values indicate the percentage of particles showing a diameter equal to or lower than the given value. Prior to all the measurements, the samples were dispersed in Milli-Q water using an Elma Transsonic Digital S T490 DH ultrasonic bath (Elma, Singen, Germany).

#### 4.5.2. Entrapment Efficiency

The encapsulation efficiency (EE) of PF was measured indirectly by quantification of the unloaded amount of PF in the dispersing agent by a reverse-phase high-performance liquid chromatography (RP-HPLC) [43]. Nanoparticles were isolated using a filtration/centrifugation procedure with Ultracel YM-100 filter (Amicon^®^ Millipore Corporation, Bedford, MA, USA) at 6000 rpm for 30 min (Sigma 301K 8 centrifuge, Osterode am Harz, Germany), with prior dilution in PBS pH 7.4 (1:20). Validation of the methodology was performed beforehand in accordance with international guidelines (EMEA, 2011) [8,44]. The EE was determined by Equation (1):

(1) $$EE\left(\%\right)=\frac{Total\;amount\;of\;PF-Free\;PF}{Total\;amount\;of\;PF}\times 100\;,$$

Samples were analysed in a Waters 1525 pump System (Waters, Milford, CT, USA) with a UV-Vis 2487 detector (Waters, Milford, CT, USA) at the wavelength λ = 235 nm using a Kromasil^®^ column (C-18, 150 × 4.6 mm, 5 µm) and methanol/glacial acetic acid 5% (70:30; *v*:*v*) as the mobile phase at the flow rate of 1 mL/min.

#### 4.5.3. Morphological Characterization

Scanning Electron Microscopy (SEM) was used to examine the morphology of the selected formulation. Samples were centrifuged at 14,000 rpm at 4 °C for 30 min. The supernatant content was discarded and the precipitate (corresponding to the PF-NLCs) was collected very carefully, and dried under vacuum for 8 days [45,46,47,48] using a vacuum desiccator. The samples were adhered to a metal tube which contained an adhesive tape where part of the dry sample was placed, and finally it was covered with carbon to generate conductivity. Images were collected using a JEOL J-7100F (Peabody, MA, USA). The SEM image was processed by Image J (1.53 t) to measure the particle size taking 22 measurements.

### 4.6. Rheological Behavior

The rheological characterization of the formulations was performed in triplicate at 25 °C using a Thermo Scientific Haake RheoStress 1 rheometer (Thermo Fisher Scientific, Karlsruhe, Germany) with a cone rotor geometry C60/2-Ti (60 mm diameter, 2° angle, 0.105 mm gap between cone-plate), coupled to a thermostatic circulator (Thermo Haake Phoenix II + Haake C25P) and operated by the software the Haake Rheowin^®^ Job Manager v 3.3 software (Thermo Electron Corporation, Karlsruhe, Germany). Haake Rheowin^®^ Data manager v. 3.3 software (Thermo Electron Corporation, Karlsruhe, Germany) was used to perform the data analyses. The viscosity and flow curves were obtained under rotational runs at 25 °C for 3 min during the ramp-up period from 0 s^−1^ to 50 s^−1^, a subsequent 1-min period at 50 s^−1^ (constant share rate period), and followed by a ramp-down period of 3 min from 50 s^−1^ to 0 s^−1^. The viscosity was determined at 50 s^−1^ after 3 days of production. The data was fitted to different mathematical models: Newton, Casson, Ostwald, Bingham Herschel-Bulkley and Cross [29].

### 4.7. Extensibility (Spreadability)

The extensibility or spreadability was determined at room temperature by placing a weight of 0.05 g of the formulation selected inside a 10 cm diameter cavity, a glass plate was positioned on top of it and pieces of increasing standard weight (5, 10, 15, 25, and 50 g) were added successively and allowed to stand on top of the glass plate for 1 min forcing the formulation to spread. The expansion in diameter was recorded as a function of the weight applied. The experiment was carried out in triplicate and fitted to a mathematical model using Graph Pad Prism^®^ software version 6.0 (GraphPad Software Inc., San Diego, CA, USA) [49].

### 4.8. Ex Vivo Permeation Study in Porcine Mucosal Tissues

To evaluate the capacity of PF to penetrate and diffuse through the mucous membranes and ophthalmic tissues, ex vivo permeation tests were conducted using different mucosal membranes from female pigs (cross Landrace × Large White, 25–30 kg), under the approval of the Ethics Committee of Animals Experimentation of the University of Barcelona. The tissues included in the study were: buccal, sublingual, nasal, vaginal mucosa, and two ophthalmic structures (sclera and cornea). The tissues were frozen to −20 °C after excision. Buccal and nasal mucosae were dermatomed (dermatome GA 630 Aesculap, Tuttlingen, Germany), at 500 µm thick slabs, the sublingual mucosa at 300 µm, and vaginal mucosa at 400 µm.

The tissues were clamped in vertical Franz cells (FDC 400, Crown Glass, Somerville, NY, USA) with a surface available diffusion area of 0.64 cm^2^ and 4.5 mL of capacity. The receptor medium was PBS pH 7.4, which was continuously in contact with the inner part of the tissue while the external side faced the donor compartment, where 500 µL of PF-NLCs-N6 was added and sealed with Parafilm^®^ to prevent evaporation. Six replicates for each tissue were included in the study. The receptor fluid was kept at 37 ± 0.5 °C under continuous magnetic stirring, except for cornea cells which were kept at 32 ± 0.5 °C. The experiments lasted 6 h during which 300 µL of the receptor compartment were collected at selected times. The same volume was replaced with fresh receptor medium after each sample collection to keep constant the volume of the receptor compartment. Samples were analysed by HPLC [8,18].

The quantification of PF retained inside the membranes and recovery required an extraction before analysis. Regarding the amount of PF retained in the tissues, we proceeded as follows: the mucous membranes and ophthalmic tissues were disassembled from the Franz cell, cleaned with a 0.05% dodecyl sulphate solution, rinsed with distilled water and weighed accurately. The tissues were pierced several times using a small needle, minced carefully, and weighed accurately. The PF was extracted with PBS pH 7.4 under sonication for 30 min in an ultrasonic water bath. The samples from drug extraction were analysed by HPLC, rendering the amount of PF extracted from the skin. The recovery was performed by incubating weighed tissues in a known concentration of PF solution in PBS pH 7.4 (Co). The incubation took place at the same temperature and with the same duration as the ex vivo experiments. After the incubation, the solution was collected, and the tissues were gently blotted and weighed again. Afterwards, the drug that had penetrated the skin was extracted by PBS as described for the retained amount. After the sonication process, the resulting solution was collected (Ex) [50]. All samples were analysed by HPLC.

We calculated the permeation parameters resulting from the permeation assays: the flux values per unit area (*J_ss_* in mg/h cm^2^), the permeability coefficients (*Kp* in cm/h) and the lag times (*Tl*) were calculated at the steady state by linear regression analysis using the Graph Pad Prism^®^ software version 6.0 (GraphPad Software Inc., San Diego, CA, USA). Stationary flux values across membranes were obtained by applying Equation (2):

(2) $$J_{ss}=\frac{Q_{t}}{A\times t}\;\;,$$

where *Q_t_* is the amount of PF which diffuses to the receptor medium (µg), *A* is the active diffusional area (cm^2^), and *t* is the time (h) of exposure per unit area. Deriving our results from the foregoing, we determined the permeability coefficient *Kp*, (cm/h) based on Equation (3):

(3) $$Kp=\frac{J_{ss}}{Co}\;\;,$$

where *J_ss_* is the flux at the steady state normalized by unit area, and *Co* is the initial drug concentration of the formulation tested and applied to the donor compartment. Partition (*P*1) and diffusion (*P*2) parameters were computed from Equations (4) and (5):

(4) $$Tl=\frac{1}{6}\times P2\;\;,$$

where *Tl* is the lag time and *P*2 the diffusion coefficient.

(5) Kp=P1×P2  ,

To predict if systemic levels of PF would be achieved after the topical application of PF-NLC-N6 to a specific surface area, we calculated the predicted plasma concentration at the steady state (*Css*) using the Equation (6):

(6) $$Css=\frac{J_{ss}\times A}{Clp}\;\;,$$

where *Css* is the concentration in plasma at the steady-state, *J_ss_* is the flux computed in this study, A is the hypothetical application area of 1 cm^2^, and *Clp* is the plasma clearance obtained from the literature; we considered two populations, the young subjects (*Clp* = 1146.60 cm^3^/h) and the elderly subjects (*Clp* = 609.00 cm^3^/h) [1,51].

Equation (7) was used to calculate the amount of PF retained in the tissue (*Qr*, µg/g/cm^2^):

(7) Qr=ExPxA×100R ,

where *Ex* (µg) is the amount of PF extracted from the tissue, *Px* (g) is the weight of the tissues exposed to the permeation, *A* (cm^2^) is the active area for diffusion and *R* is the recovery of the PF from the tissue, expressed as a percentage.

### 4.9. Stability Studies

Short-term physical stability was assessed after 1, 30, and 60 days analysing light backscattering (BS) profiles by using the Turbiscan^®^Lab (Formulaction Co., L’Union, France). For this purpose, a cylindrical glass measurement cell was filled with 20 mL of PF-NLCs-N6 stored at 25 °C for two months. The radiation source was a pulse near infrared light (λ = 880 nm) and it was received by backscattering detectors at an angle of 45° from the incident beam due to the opacity of the formulation.

Additionally, morphometric parameters (Z-Ave, PI and ZP) and EE were also measured at 25 °C and 4 °C, monitored for 24 h and after 7, 14, 30, 60 and 90 days to evaluate any potential changes.

### 4.10. In Vitro Ocular Tolerance Study: Hen’s Egg Test on the Chorioallantoic Membrane (HET-CAM)

In vitro ocular tolerance was assessed using the HET-CAM test to ensure that the formulation of PF-NLC-N6 was non-irritating when administered as eye drops. 300 μL of the formulation studied was applied on the chorioallantoic membrane of a fertilized chicken egg and monitored for 5 min after the application of the formulation. The following phenomena were considered: irritation, coagulation, and haemorrhaging.

The development of the test was carried out using 3 eggs for each group (formula PF-NLCs-N6, negative control (0.9% NaCl), positive control (NaOH 0.1 N)). The ocular irritation index (*OII*) was calculated by the sum of the scores of each injury or discomfort according to the following expression (Equation (8)):

(8) $$OII=\frac{\left(301-H\right)\times 5}{300}+\frac{\left(301-V\right)\times 7}{300}+\frac{\left(301-C\right)\times 9}{300}\;,$$

where *H*, *V* and *C* are times (in seconds) until the start of haemorrhaging (*H*), vasoconstriction (*V*) and coagulation (*C*), respectively. The formulations were classified according to the following scores: OII ≤ 0.9 non-irritating; 0.9 < OII ≤ 4.9 weakly irritating; 4.9 < OII ≤ 8.9 moderately irritating; 8.9 < OII ≤ 21 irritating [52,53].

## 5. Conclusions

A nanostructured lipid carrier has been developed by means of QbD, which allowed the influence of the components in the formulation to be understood. It was seen that the factorial design played a key role in optimizing the formulation. Finally, the optimized nanoparticles were tested on ex vivo porcine mucosal tissues (buccal, sublingual nasal, vaginal, cornea and sclera) to evaluate their capacity to diffuse the tissues and, in turn, their potential to treat different inflammatory conditions in mucosal tissues with a topical approach. High permeation and high retention were observed in all the tissues tested. In particular, the highest permeation was found on the cornea; and PF was mostly retained in the sublingual mucosa. The optimized nanoparticles exhibit suitable characteristics for the topical delivery on the tested mucosae, and they were shown to be safe for the ocular route since no irritant effects were observed in the HET-CAM test. Furthermore, the predicted concentration at the steady-state was below the therapeutic concentration of PF in plasma, and this may result in a local anti-inflammatory and analgesic effect on damaged mucosae.

## Figures and Tables

**Table 1:** Solubility of pranoprofen in different solid lipids and liquid lipids.

	0.1% PF		0.5% PF		1.0% PF	
Lipid Raw Materials	Solubility	Appearance	Solubility	Appearance	Solubility	Appearance
SOLID LIPIDS						
Stearic acid	−	Semi-solid	−	Semi-solid	−	Semi-solid
Precifac^®^ ATO	−	Hard	−	Hard	−	Hard
Compritol^®^ 888 ATO	−	Soft	−	Soft	−	Soft
Lanette^®^18	+	Hard	+	Hard	−	Hard
Precirol^®^ATO 5	+	Hard	+	Hard	−	Hard
Gelucire^®^ 44	+	Semi-solid	−	Semi-solid	−	Semi-solid
Geleol^®^	−	Soft	−	Soft	−	Soft
LIQUID LIPIDS						
Isopropyl myristate	+	Clear solution	−	Precipitation	−	Precipitation
Plantacare oil	+	Clear solution	+	Clear solution	−	Precipitation
Miglyol^®^ 812	+	Clear solution	−	Precipitation	−	Precipitation
Rose mosqueta oil	+	Clear solution	−	Precipitation	−	Precipitation
Jojoba oil	+	Clear solution	−	Precipitation	−	Precipitation
Castor oil	+	Clear solution	+	Clear solution	−	Precipitation
LAS (PEG-8 Caprylic/Capric Glycerides)	+	Clear solution	+	Clear solution	+	Clear solution

+ pranoprofen is soluble; − pronoprofen is insoluble.

**Table 2:** Solubility of pranoprofen in mixtures of lipids.

	1.5% PF		2.0% PF		3.0% PF	
Physical Mixtures of Lipids	Solubility	Appearance	Solubility	Appearance	Solubility	Appearance
LAS:Castor oil (50:50)	+	Clear solution	−	Precipitation	−	Precipitation
LAS:Castor oil (60:40)	+	Clear solution	−	Precipitation	−	Precipitation
LAS:Castor oil (75:25)	+	Clear solution	+	Clear solution	−	Precipitation
Precirol^®^ATO 5:(LAS-Castor oil (75:25)) 40:60	+	Semi-solid	+	Semi-solid	−	Semi-solid
Precirol^®^ATO 5:(LAS-Castor oil (75:25)) 50:50	+	Hard	+	Hard	−	Hard
Precirol^®^ATO 5:(LAS-Castor oil (75:25)) 60:40	+	Hard	−	Hard	−	Hard

+ pranoprofen is soluble; − pronoprofen is insoluble.

**Table 3:** Factors and their corresponding coded levels of experimental design.

Factors	−1.68	−1	0	1	1.68
cSL/LL (%)	43.2	50	60	70	76.8
cPF (%)	0.16	0.5	1	1.5	1.84
cTW (%)	2.16	2.5	3	3.5	3.84

cSL/LL: concentration of solid lipid concerning liquid lipid; cPF: concentration of PF, cTW: concentration of TW 80.

**Table 4:** Independent and dependent variables of the 2^3^ start central composite rotatable factorial design factors.

Formulations	Independent Variables						Dependent Variables			
	cSL/LL		cPF		cTW		Z-Ave (nm)	Pl	ZP (mV)	EE (%)
PF-NLCs-N1	1	70	1	1.5	1	3.5	219.3	0.305	−10.70	98.30
PF-NLCs-N2	0	60	0	1	−1.68	2.16	329.5	0.373	−10.56	98.50
PF-NLCs-N3	−1	50	−1	0.5	−1	2.5	449.1	0.452	−12.48	96.90
PF-NLCs-N4	−1.68	43.2	0	1	0	3	345.1	0.365	−8.60	98.54
PF-NLCs-N5	0	60	0	1	0	3	283.5	0.287	−10.20	97.21
**PF-NLCs-N6**	**−1**	**50**	**1**	**1.5**	**−1**	**2.5**	**214.2**	**0.266**	**−10.81**	**98.33**
PF-NLCs-N7	1	70	−1	0.5	−1	2.5	267.4	0.478	−8.56	94.30
PF-NLCs-N8	0	60	−1.68	0.16	0	3	345.8	0.499	−9.60	93.36
PF-NLCs-N9	−1	50	−1	0.5	1	3.5	321.5	0.419	−10.00	98.15
PF-NLCs-N10	1.68	76.8	0	1	0	3	290.9	0.363	−9.99	98.49
PF-NLCs-N11	0	60	1.68	1.84	0	3	327.9	0.267	−8.96	91.54
PF-NLCs-N12	0	60	0	1	1.68	3.84	359.7	0.486	−8.34	93.58
PF-NLCs-N13	0	60	0	1	0	3	272.8	0.294	−10.50	97.86
PF-NLCs-N14	−1	50	1	1.5	1	3.5	252.4	0.286	−10.34	97.22
PF-NLCs-N15	1	70	1	1.5	−1	2.5	257.1	0.295	−9.84	97.15
PF-NLCs-N16	1	70	−1	0.5	1	3.5	387.8	0.34	−10.3	90.1

cSL/LL: concentration of solid lipid concerning liquid lipid; cPF: concentration of PF; cTW: concentration of TW 80; Z-Ave: mean particle size; PI: poly dispersity index; ZP: zeta potential and EE: encapsulation efficiency.

**Table 5:** Summary of importance of principal components.

	Importance of Components						
	PC1	PC2	PC3	PC4	PC5	PC6	PC7
SD	1.5804	1.2667	1.0206	0.9006	0.8158	0.4872	0.3770
Prop V	0.3568	0.2292	0.1488	0.1159	0.0951	0.0339	0.0203
Cum P	0.3568	0.5860	0.7348	0.8507	0.9458	0.9797	1.0000

SD: standard deviation; Prop V: proportion of variance, and Cum P: cumulative proportion.

**Table 6:** Correlation matrix of variables and principal components.

	Importance of Components						
	PC1	PC2	PC3	PC4	PC5	PC6	PC7
cLS/LL	−0.01	0.51	−0.79	0.15	−0.25	0.16	0.04
cPF	0.83	0.31	0.18	0.12	0.26	0.25	−0.19
cTW	−0.20	0.56	0.57	0.18	−0.53	0.04	0.00
Z-Ave (nm)	−0.79	−0.29	0.16	0.34	0.21	0.30	0.11
Pl	−0.86	−0.14	−0.10	−0.37	−0.12	0.09	−0.25
ZP (mV)	−0.23	0.72	0.13	−0.56	0.28	0.06	0.13
EE(%)	0.58	−0.56	0.06	−0.42	−0.34	0.22	0.10

cLS/LL: concentration of solid lipid concerning liquid lipid; cPF: concentration of pranoprofen; cTW: concentration of Tween 80; Z-Ave: mean particle size; PI: polydispersity index; ZP: zeta potential and EE: encapsulation efficiency.

**Table 7:** Correlation matrix of variables and principal components.

	Degrees of Freedom	Pillai	Approx F Statistic	Degrees of Freedom (Numerator)	Degrees of Freedom (Denominator)	Pr (>F)
cLS/LL	1	0.26843	0.8256	4	9	0.54092
cPF	1	0.69140	5.0409	4	9	0.02071 *
cTW	1	0.12866	0.3322	4	9	0.84961
Residuals	12	-	-	-	-	0.54092

Significance codes: * = 0.01.

**Table 8:** Physicochemical stability of PF-NLCs-N6 at 25 °C (*n* = 3), monitored by the evaluation of any change in the following parameters: mean particle size, polydispersity index, zeta potential and encapsulation efficiency.

Time (days)	Z-Ave (nm) ± SD	PI ± SD	ZP (mV) ± SD	EE (%)
1	218.09 ± 3.97	0.297 ± 0.03	−10.13 ± 0.11	98.02
7	232.07 ± 5.43	0.299 ± 0.06	−10.01 ± 0.09	97.46
14	267.97 ± 3.93	0.304 ± 0.03	−10.00 ± 0.07	97.54
30	300.72 ± 8.76	0.312 ± 0.05	−9.89 ± 0.16	96.68
60	318.21 ± 5.12	0.329 ± 0.06	−9.53 ± 0.20	96.03
90	326.07 ± 4.88	0.344 ± 0.05	−9.48 ± 0.09	96.52

**Table 9:** Physicochemical stability of PF-NLCs-N6 at 4 °C (*n* = 3), monitored by the evaluation of any change in the following parameters: mean particle size, polydispersity index, zeta potential and encapsulation efficiency.

Time (days)	Z-Ave (nm) ± SD	PI ± SD	ZP (mV) ± SD	EE (%)
1	220.14 ± 4.92	0.288 ± 0.04	−10.25± 0.10	97.99
7	242.10 ± 3.63	0.301 ± 0.04	−10.16± 0.07	98.45
14	256.24 ± 4.09	0.299 ± 0.05	−9.90 ± 0.09	97.73
30	284.15 ± 5.09	0.309 ± 0.04	−9.76 ± 0.12	97.12
60	303.16 ± 5.94	0.316 ± 0.04	−9.66 ± 0.15	97.23
90	315.02 ± 4.83	0.327 ± 0.06	−9.52 ± 0.08	96.81

**Table 10:** The results of the permeation parameters of PF at 6 h from the selected formula in the different mucosal tissues tested, *n* = 6 each. The results are reported as the median (maximum and minimum): Flux (Jss), lag time (Tl), partition coefficient P1, diffusion coefficient P2, permeability coefficient (Kp).

	Buccal	Sublingual	Nasal	Vaginal	Sclera	Cornea
Jss (µg/h)	2.46	2.34	3.26	2.59	2.57	5.02
	(2.14–2.96)	(2.14–2.63)	(2.45–5.11)	(1.96–2.84)	(2.15–2.83)	(4.15–6.32)
Tl (h)	4.10	3.67	1.18	0.94	3.66	0.09
	(3.92–4.37)	(3.14–4.99)	(0.83–1.94)	(0.51–1.31)	(2.41–4.93)	(0.04–0.11)
P2 (10^−1^ h^–1^)	0.41	0.45	1.41	3.26	0.45	18.52
	(0.38–0.42)	(0.33–0.53)	(0.86–2.01)	(1.27–1.77)	(0.33–0.69)	(15.15–41.67)
P1 (10^−3^ cm)	0.16	0.13	0.06	0.03	0.15	0.007
	(1.30–2.00)	(1.00–2.10)	(0.30–1.60)	(0.10–0.60)	(0.80–2.20)	(0.03–0.11)
Kp (10^−4^ cm/h)	0.65 (0.56–0.78)	0.61 (0.56–0.69)	0.85 (0.64–1.34)	0.68 (0.51–0.74)	0.67 (0.56–0.74)	1.32 (1.09–1.66)

**Table 11:** The results of the pranoprofen recovery with phosphate-buffered saline (PBS), expressed as a percentage of the recovery, and its relative standard derivation (%RSD); *n* = 3 each.

Membrane	Recovery (%)	RSD (%)
Buccal	17.03	1.60
Sublingual	8.72	0.70
Nasal	16.03	1.36
Vaginal	12.65	0.71
Sclera	12.97	1.15
Cornea	15.01	1.34

**Table 12:** The results of the retained amount (Qr) of pranoprofen at 6 h from the selected formula (PF-NLCs-N6) in the tissues (buccal, sublingual nasal and vaginal, cornea and sclera), *n* = 6 each. Values are reported as median (maximum and minimum).

Membrane	Qr (µg/cm^2^/g)
Buccal	1488.52
	(1227.62–1563.24)
Sublingual	3198.92
	(3005.43–3321.72)
Nasal	1879.92
	(1765.70–1990.44)
Vaginal	591.01
	(437.20–625.30)
Sclera	1842.73
	(1711.32–1897.90)
Cornea	745.09
	(623.72–804.57)

**Table 13:** Predicted plasma levels of PF at the steady-state (Css) for the young and elderly populations, obtained from the selected formulation applied on 1 cm^2^ of porcine mucous membrane (buccal, sublingual, nasal and vaginal) and ophthalmic tissues (sclera and cornea). Data are reported as median (min-max).

Membrane	Young Subject Css (ng/mL)	Elderly Subject Css (ng/mL)
Buccal	2.14	4.04
	(1.87–2.58)	(3.51–4.87)
Sublingual	2.04	3.84
	(1.87–2.29)	(3.51–4.32)
Nasal	2.84	5.35
	(2.14–4.46)	(4.02–8.39)
Vaginal	2.26	4.25
	(1.71–2.48)	(3.22–4.67)
Sclera	2.24	4.22
	(1.87–2.47)	(3.53–4.65)
Cornea	4.38	8.24
	(3.62–5.51)	(6.81–10.38)

## Data Availability

The data presented in this study are available on request from the corresponding author.
